# Cardiac interaction between mother and infant: enhancement of heart rate variability

**DOI:** 10.1038/s41598-019-56204-5

**Published:** 2019-12-27

**Authors:** Ayami Suga, Maki Uraguchi, Akiko Tange, Hiroki Ishikawa, Hideki Ohira

**Affiliations:** 1 Unicharm Corporation, 769-1602 Kagawa, Japan; 2 Department of Psychology, Nagoya University, 464-8601 Nagoya, Japan

**Keywords:** Physiology, Human behaviour

## Abstract

The vagal activity of infants is represented by heart rate variability (HRV) and associated with both growth and socioemotional development. The enhancement of an infant’s vagal tone activity might be beneficial for development. This study explored whether HRV in infants aged 3–8 months can be enhanced by influencing HRV in mothers (40 dyads). The power of the low frequency (LF) component of maternal HRV was facilitated using slow-paced breathing. We investigated whether the change in maternal HRV affected the LF component in infants held by their mothers. In older infants (*N* = 14, 6–8 months) the LF power showed an increase during maternal paced breathing, whereas a delayed increase occurred after termination of maternal paced breathing in younger infants (*N* = 16, 3–5 months). These results show that the effects of maternal cardiac activity on the infant’s HRV are age-dependent. This age-dependent reactivity of the infant’s HRV could be due to the development of the inner model in infants which regulates physiological functions, including cardiac activity. This finding might help develop efficient methods for enhancing vagal nerve activity in infants.

## Introduction

Heart rate variability (HRV) is the fluctuation of intervals between heartbeats and has been utilised to estimate vagal nerve activity^1^, which reflects a core regulation mechanism of the brain and body. A decrease in HRV is linked to vulnerability to stress whereas an increase in HRV represents physical and mental adaptability^1^. Previous studies reported that HRV in the foetal, neonatal and infant periods could predict various aspects of development. The development of HRV in foetuses is related to language skills and psychological development in early childhood^2^. The vagal activity of infants, represented by HRV, is associated with both growth and socioemotional development^3^. These findings led us to infer that the enhancement of an infant’s vagal tone activity might be beneficial for his/her development.

In previous studies, the interactions of heartbeats between mothers and foetuses were observed^4,5^. The cardiac interaction between mothers and infants was demonstrated by examining the reactivity of HRV in infants to fluctuations in their mothers’ respiratory sinus arrhythmia (RSA), which is one of the components of HRV^6^. In that study, mothers conducted paced breathing while their infants lay on their body with skin-to-skin contact. The RSA in infants during the first 2 months of age can be elevated by increasing their mothers’ RSA. However, at 3 months of age, the infants’ RSA deviated from their mothers’ RSA. The authors stated that the reduction of physiological adaptation between 8 and 12 weeks might reflect the shift from an intra-uterine to an extra-uterine condition.

This result suggests the phenomenon of cardiac synchronisation between mother and infant might be dependent on the infants’ age. In neonatal studies, the respiratory control of premature infants has been influenced by the caregivers’ cardiac rhythms during skin-to-skin contact^7^. Such skin-to-skin contact has a favourable effect on parasympathetic nerve activities in premature infants and mothers^8^. Furthermore, in 3-month old infants, vocal synchrony between mother and infant during face-to-face interactions coordinates their heart rhythms^9^.

Nevertheless, developmental changes in the physiological interaction of cardiac activity in mother and infant after 3 months have not been clarified. During the period of rapid development of motor functions that enable crawling and walking, specifically at 3–8 months old, the prominent development of the autonomic nerve supports such motor functions, reflected by an increase in infant HRV, which is parallelly observed^10,11^. Considering this finding, the characteristics of cardiac interactions between mother and infant might change during such a period.

We explored whether the HRV in infants at 3–8 months old, during the rapid development of their motor functions and the autonomic nerve control, is influenced by HRV in mothers as reported by Van Puyyelde^6^, after the intra-uterine effect disappears. To achieve this aim, we manipulated mothers’ respiration to increase their HRV, in a situation where they kept physical contact with their infants. It was reported that breathing at a rate of approximately six cycles per minute (cpm) increased the low frequency (LF) component power of HRV^12,13^. HRV is influenced by two primary physiologic fluctuations: RSA and the Mayer waves in blood pressure. RSA is a naturally occurring variation in the heart rate that occurs during a breathing cycle, where the heart rate increases during inspiration and decreases during expiration. This fluctuation usually occurs at a frequency of 3–4 seconds. The respiration rate of a healthy adult woman is in the range of 14–18 breaths per minute^14,15^, making natural RSA occurring in the frequency range of 3–4 seconds. Mayer waves in blood pressure occur at a frequency of 0.1 Hz or a 10-second periodicity. With a breathing rate of 6 cpm or a 10-second periodicity, it is thought that RSA and the Mayer wave become synchronised and can enhance HRV due to their resonance^16^.

We predicted that the HRV of infants hugged by their mothers would be enhanced by increasing the mothers’ HRV through paced respiration. The influence of the mothers’ HRV on the HRV in infants might be mediated by the physical synchronisation of cardiac activities between mother and infant, and the magnitude of the influence might depend on the infant’s age. Specifically, we explored whether the influence of the mothers’ HRV on the infants’ HRV was different between relatively older infants (6–8 months old) and younger infants (3–5 months old). Furthermore, the directions of HRV influences between mother and infant were estimated by calculating transfer entropy (TE) of heartbeats between mothers and infants^17,18^.

## Results

Considering the wide individual differences in the LF, the high frequency (HF) components of HRV in mothers and infants and the TE between mother and infant heartbeats at each baseline, we conducted a 3-way repeated measures analyses of variance (ANOVA) (developmental stage (older vs. younger) × condition (natural- breathing vs. paced- breathing) × period (pre-rest, respiration 0–5 min, respiration 5–10 min, respiration 10–15 min and post-rest) to assess the changes in those indices from the baselines. Although we mainly focused on the LF component of HRV to measure the effects of paced respiration on HRV^12^, we also examined the HF power and LF/HF ratio of HRV.

**Figure 1:**
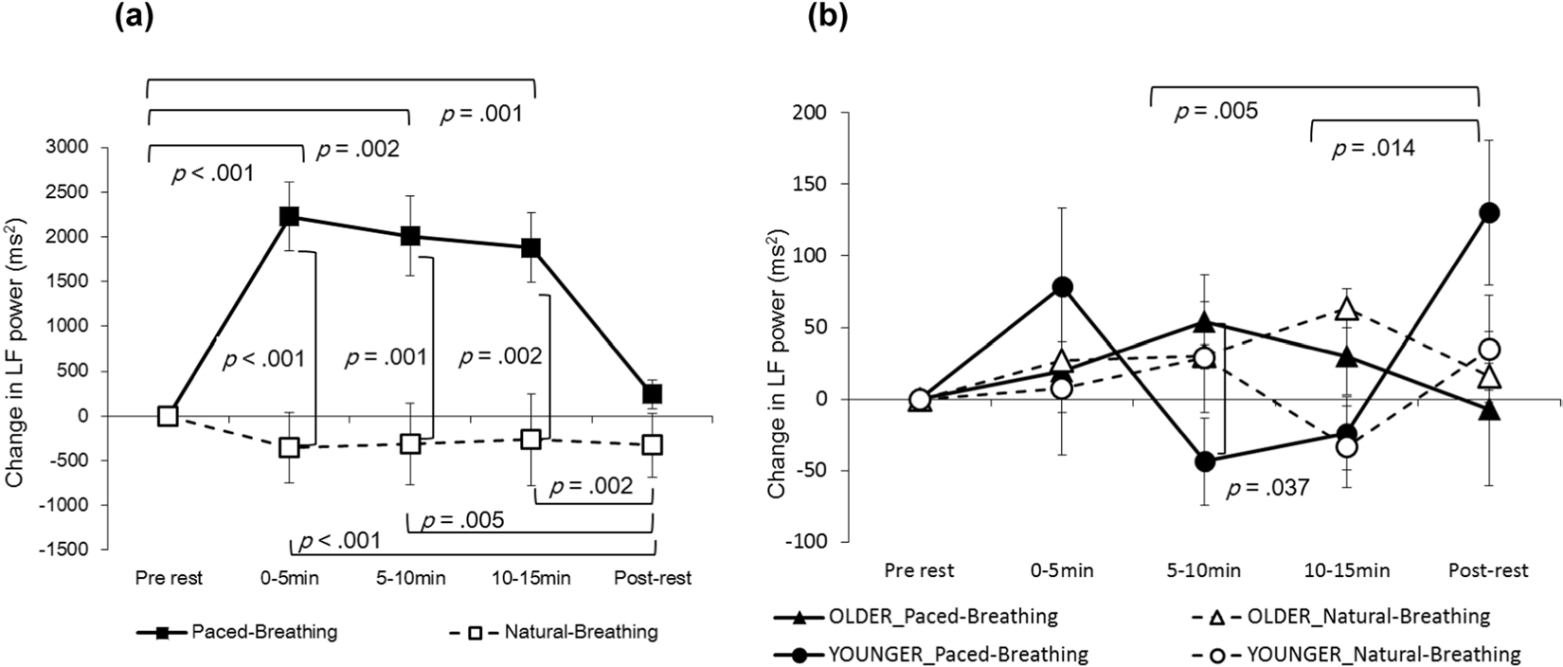
Change in LF power of HRV. Left (**a**) in mothers (*n* = 22), right (**b**) in infants (*n* = 30). Error bars indicate standard errors.

**Figure 2:**
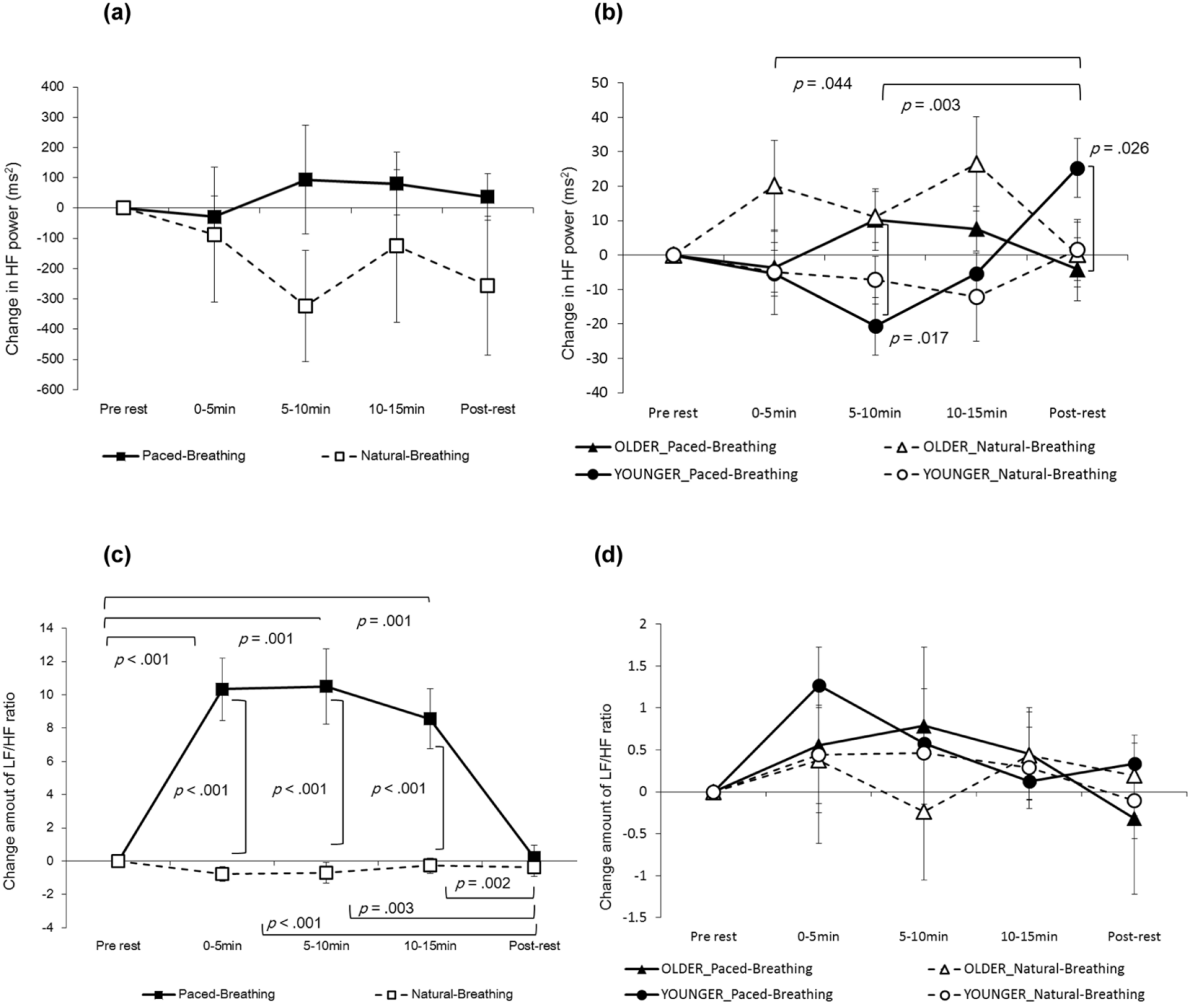
Change in HF power of HRV and change amount of LF/HF ratio. Left (**a**) in mothers’ HF power (*n* = 22), right (**b**) in infants’ HF power (*n* = 30). Left (**c**) in mothers’ LF/HF ratio (*n* = 22), right (**d**) in infants’ LF/HF ratio (*n* = 30). Error bars indicate standard errors.

The interaction between the condition and period was significant for the LF power in mothers, (*F* (4, 84) = 9.8639, *p* < 0.001, partial *η*^2^ = 0.320; Fig. 1(a)). In the paced-breathing condition, the LF power during the periods of 0–5 min, 5–10 min and 10–15 min was significantly higher than at the pre-rest period (*p* < 0.001, *p* = 0.002 and *p* = 0.001, respectively) as well as during the post-rest period (*p* < 0.001, *p* = 0.005 and *p* = 0.002, respectively). During the periods of 0–5 min, 5–10 min and 10–15 min, the LF power in the paced-breathing condition was significantly higher than in natural-breathing condition (*p* < 0.001, *p* = 0.001 and *p* = 0.002, respectively). The HF power in mothers showed neither significant main effects nor interactions (Fig. 2(a)).

In relation to the change in the LF/HF ratio in mothers, the interaction between condition and period was significant (*F* (2.655, 55.755) = 14.085, *p* < 0.001, partial *η*^2^ = 0.401; Fig. 2(c)). In the paced-breathing condition, the LF/HF ratio for power in periods of 0–5 min, 5–10 min and 10–15 min was significantly higher than in the pre-rest period (*p* < 0.001, *p* = 0.001 and *p* = 0.001, respectively) as well as in the post-rest period (*p* < 0.001, *p* = 0.003 and *p* < 0.002, respectively). In periods of 0–5 min, 5–10 min and 10–15 min, the LF/HF ratio in the paced-breathing condition was significantly higher than in the natural-breathing condition (*p* < 0.001 in all conditions).

The LF power in infants indicated that the interaction between condition, period and developmental stage was significant (*F* (4, 112) = 2.757, *p* = 0.031, partial *η*^2^ = 0.090; Fig. 1(b)). We examined a simple interaction effect between period and developmental stage for each condition. In natural-breathing conditions, the simple interaction was not significant (*F* (4,112) = 1.477, *p* = 0.214, partial *η*^2^ = 0.050). On the other hand, in paced-breathing conditions, the simple interaction was significant (*F* (4,112) = 4.586, *p* = 0.002, partial *η*^2^ = 0.141), suggesting that the LF power of the younger infants was significantly lower than that of the older infants during the 5–10 min period (*p* = 0.037). Additionally, in younger infants only, the LF power during the post-rest period was significantly higher than during the periods of 5–10 min and 10–15 min (*p* = 0.005 and *p* = 0.014, respectively). The simple interaction effect between period and condition was not significant at either developmental stage.

The HF power in infants indicated that the interaction between condition, period and developmental stage was close to significant (*F* (4,112) = 2.369, *p* = 0.057, partial *η*^2^ = 0.078; Fig. 2(b)). In natural-breathing conditions, the simple interaction was close to significant (*F* (4, 112) = 2.435, *p* = 0.051, partial *η*^2^ = 0.080). In paced-breathing conditions, the simple interaction was significant (*F* (4,112) = 6.949, *p* < 0.001, partial *η*^2^ = 0.199), suggesting that HF power in older infants was significantly higher than that in younger infants during the 5–10 min period (*p* = 0.017). The younger infants’ HF power was significantly higher than the older infants during the post- rest period (*p* = 0.026). In younger infants only, the HF power during the post-rest period was significantly higher than during the 5–10 min and 10–15 min periods (*p* = 0.044 and *p* = 0.003, respectively). The simple interaction effect between period and condition was not significant at either developmental stage. The change in infants’ LF/HF ratios demonstrated no significant main effects or interactions (Fig. 2(d)).

**Figure 3:**
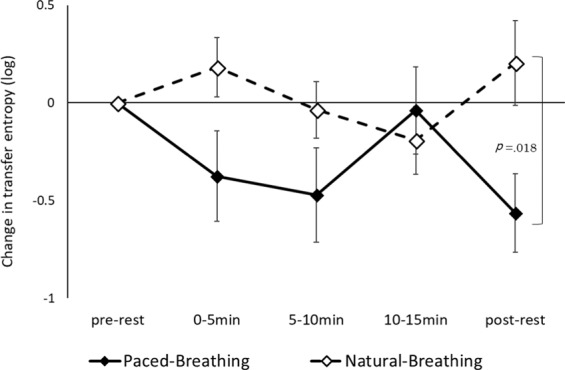
Change in transfer entropy from mothers to infants (19 dyads). Error bars indicate standard errors.

The interaction between condition and period was significant for the logged values of TE from mothers to infants (*F* (4, 68) = 3.103, *p* = 0.021, partial *η*^2^ = 0.154; Fig. 3), suggesting that the logged values of TE in paced-breathing conditions was significantly lower than during natural-breathing conditions during the post-rest period (*p* = 0.018). No other effect was significant. A repeated measure ANOVA measuring changes in the logged values of TE from infants to mothers showed no significant main effects or interactions.

A Pearson’s correlation analyses of the mothers’ LF power, the infants’ LF power and the infants’ HF power were performed to explore the associations between mothers’ and infants’ cardiac activities in the paced-breathing conditions (Table 1). The LF power of mothers during the respiration 0–5 min period and 10–15 min period was strongly related to the LF power of infants during the respiration 10–15 min period for the older group (*r* = 0.863, *p* = 0.006 and *r* = 0.779, *p* = 0.023). There was no correlation between the LF power of mothers and the HF power of infants. For older infants, the LF power during the respiration 0–5 min period was strongly related to the HF power during the respiration 0–5 min period (*r* = 0.715, *p* = 0.046). For younger infants, the LF power during the 5–10 min period of respiration was strongly related to the HF power of post-rest (*r* = 0.691, *p* = 0.018).

**Table 1:** Pearson’s correlation coefficient between mothers’ LF, infants’ LF and infants’ HF in the paced-breathing condition (19 dyads).

	Variables		1	2	3	4	5	6	7	8	9	10	11
1	Mothers’ LF	Younger	—										
	0–5 min	Older	—										
2	Mothers’ LF	Younger	0.638^*^	—									
	5–10 min	Older	0.700^†^	—									
3	Mothers’ LF	Younger	0.688^*^	0.955^**^	—								
	10–15 min	Older	0.919^**^	0.573	—								
4	Infants’ LF	Younger	−0.297	−0.247	−0.209	—							
	0–5 min	Older	0.634^†^	0.597	0.717^*^	—							
5	Infants’ LF	Younger	0.441	0.213	0.207	0.473	—						
	5–10 min	Older	0.639^†^	0.340	0.665^†^	0.840^**^	—						
6	Infants’ LF	Younger	0.009	−0.142	−0.099	0.563^†^	0.636^*^	—					
	10–15 min	Older	0.863^**^	0.419	0.779^*^	0.258	0.367	—					
7	Infants’ LF	Younger	0.153	−0.030	0.034	0.825^**^	0.836^**^	0.656^*^	—				
	Post-rest	Older	0.541	0.241	0.298	0.055	0.083	0.584	—				
8	Infants’ HF	Younger	0.291	0.332	0.287	0.309	0.555^†^	0.554^†^	0.399	—			
	0–5 min	Older	0.565	0.554	0.662^†^	0.715^*^	0.431	0.211	0.062	—			
9	Infants’ HF	Younger	0.208	−0.118	−0.136	0.188	0.514	0.650^*^	0.310	0.797**	—		
	5–10 min	Older	0.498	0.090	0.624^†^	0.380	0.683^†^	0.457	−0.184	0.126	—		
10	Infants’ HF	Younger	0.202	0.338	0.309	0.375	0.563^†^	0.536^†^	0.440	0.356	0.354	—	
	10–15 min	Older	0.462	−0.011	0.617	0.356	0.640^†^	0.524	−0.242	0.169	0.896^**^	—	
11	Infants’ HF	Younger	0.560^†^	0.013	0.021	0.003	0.691^*^	0.379	0.458	0.393	0.496	−0.079	—
	Post-rest	Older	−0.168	−0.477	0.072	−0.424	−0.342	0.156	−0.194	−0.312	0.327	0.312	—

The standard for the error analysis was 5%. **p < 0.01, *p < 0.05, ^†^*p* < 0.10.

**Figure 4:**
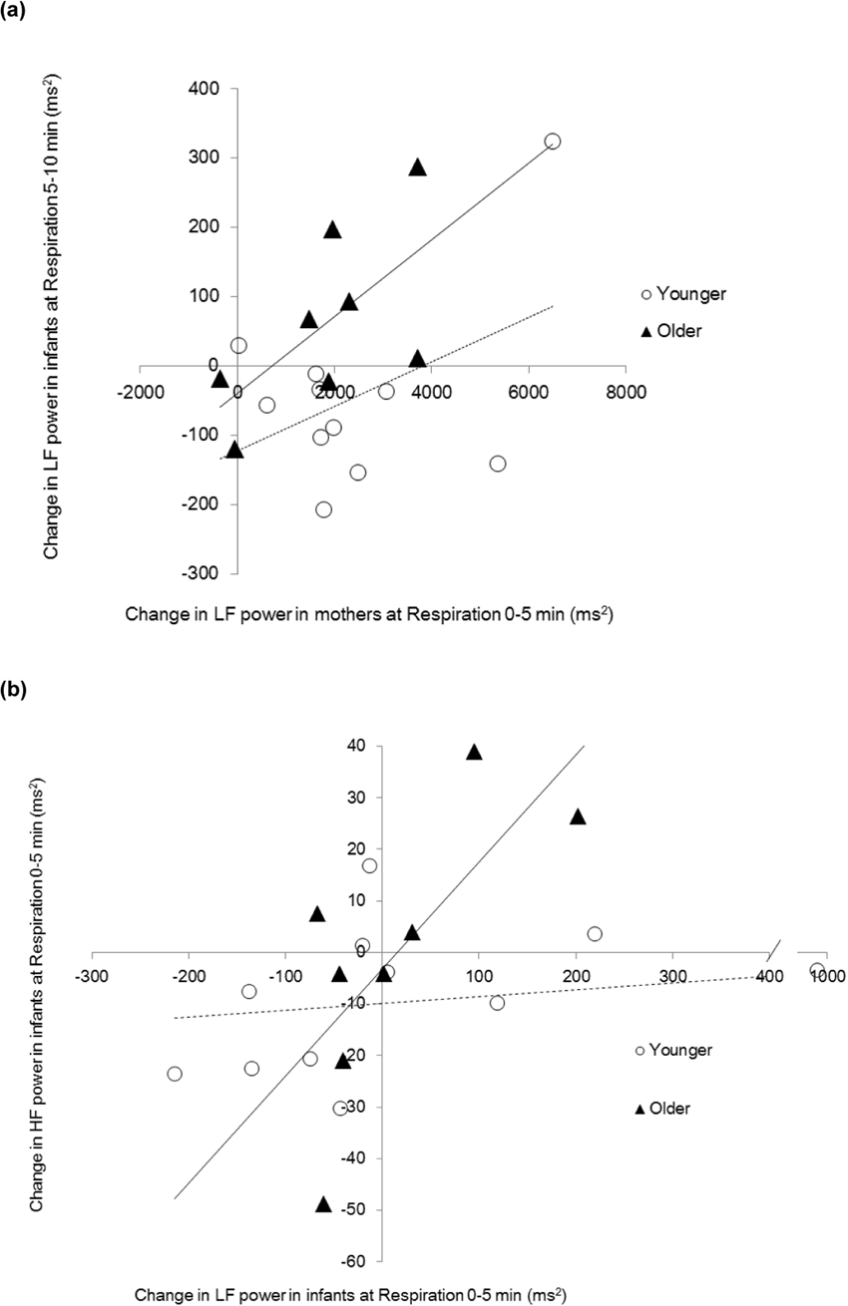
(**a**) Association between the change in LF power of infants and the change in LF power of mothers (19 dyads). (**b**) Association between the change in HF power and the change in LF power of infants. (*n* = 19) Solid line; regression line for older infants, broken line; regression line for younger infants.

On the basis of the correlational analyses, a regression analysis was performed using the mothers’ LF changes and developmental stage as independent variables and the infants’ LF changes as a dependent variable. The multiple linear regressions were calculated to predict the change in infants’ LF power in relation to a change in mothers’ LF power and the developmental stage during each period of the experiment. The best fitting model for predicting the change in infants’ LF power during the respiration 5–10 min period was a linear combination of the LF power of mothers during the respiration 0–5 min period and developmental stage (*F* (2, 16) = 4.521, *p* = 0.028, with an *R*^2^ of 0.361; Table 2, Fig. 4a). Both the LF power of mothers and developmental stage had significant effects (*B* = 0.039, *SE* = 0.017, *p* = 0.032 and *B* = 129.352, *SE* = 57.107, *p* = 0.038). The addition of the interaction variable did not significantly improve prediction (Δ*R*^2^ = 0.016, Δ*F* = 0.393, *p* = 0.540).

**Table 2:** Coefficients in the regression analysis. The effects of the mothers’ HRV on the infants’ HRV in paced-breathing conditions (19 dyads).

Independent variables		Step 1			Step 2		
		*B*	*SE B*	*β*	*B*	*SE B*	*β*
Step 1	Change in mothers’ LF power in respiration 0–5 min	0.039*	0.017	0.477*	0.042	0.018	0.511*
	Developmental stages	129.352*	57.107	0.460*	133.306	58.563	0.474*
Step 2	Change in mothers’ LF × Developmental stage	—	—	—	0.023	0.034	0.132
*R*^2^		0.361*			0.377^†^		

Note: Dependent variable is the change in the LF power in the infants in respiration 5–10 min. The standard for the error analysis was 5%. **p< 0.01, *p< 0.05, ^†^*p*< 0.10.

Significant associations between mothers’ LF power and infants’ HF power were not found in either the older or younger groups.

A similar regression analysis was performed to track associations between infants’ LF change and HF change during each developmental stage. Developmental stage significantly moderated the association between the change in LF power of infants during the respiration 0–5 min period and the change in HF power during the respiration 0–5 min period (Δ*R*^2^ = 0.291, Δ*F* = 7.595, *p* = 0.015; Table 3). A simple slope test demonstrated that LF power was associated positively with HF power in the older group (*B* = 0.194, *SE* = 0.064, *p* = 0.008, Fig. 4b), while no significant association was observed in the younger group (*B* = −0.003, *SE* = 0.019, *p* = 0.856).

**Table 3:** Coefficients in the regression analysis. Effects of infants’ LF on the infants’ HF in the paced-breathing condition (*n* = 19).

Independent variables		Step 1			Step 2		
		*B*	*SE B*	*β*	*B*	*SE B*	*β*
Step 1	Change in infants’ LF power in respiration 0–5 min	0.024	0.019	0.294	0.095*	0.030	1.175**
	Developmental stages	10.062	9.395	0.250	14.900^†^	8.099	0.370^†^
Step 2	Change in infants’ LF × Developmental stage	—	—	—	0.195**	0.071	1.031*
*R*^2^		0.135			0.426*		

Note: The dependent variable is the change in the HF power in the infants in respiration 0–5 min.

The standard for the error analysis was 5%. **p < 0.01, *p < 0.05, ^†^*p* < 0.10.

## Discussion

The maximum heart rate oscillations at respiratory frequency occurred at approximately 0.1 Hz (6 bpm)^16^. The respiration-induced changes in HRV may function as a positive feedback loop, spiralling further increases in HRV due to feedback from the heart to the central nervous system through the vagal afferent system. In this study, a robust increase in the power of the LF component and the LF/HF ratio of HRV, as well as no change in the HF power of HRV, in mothers performing paced breathing were observed. These results are consistent with previous findings^19,20^. These findings verified the validity of the experimental manipulation performed in this study.

The influence of mothers’ enhanced HRV while performing paced-breathing on their infants’ HRV depended on the infant’s age; the increase in LF power in older infants was greater than in younger infants. The multiple regression analysis indicated that the LF power of infants increased according to an increase in the mothers’ LF power, and this tendency was greater in older infants than in younger infants. The TE analysis suggested that the association of HRV between mothers and older infants was due to the beat by beat influence of the mothers’ cardiac activity on the infants’ cardiac activity. The results indicating that the influences of the infants’ cardiac activity on the mothers’ cardiac activity were relatively small seems rational, considering that infants have much smaller hearts than their mothers, thus their physical impact should be smaller. The heart rate of an adult can be easily synchronised to an external sequential pulse such as sound and light, even when the stimulus is weak, via physical entrainment^21^. Our finding suggests that such mechanisms should be developed in infants at least by 8 months old.

Interestingly, while the increase in older infants’ LF power due to the influence of mothers’ LF power quickly returned to the baseline just after the termination of paced breathing, the LF power in younger infants showed delayed increase during the post-rest period when mothers were no longer performing paced breathing. Also, the correlation analysis found no correlation between the mothers’ LF power and the younger infants’ LF power during the paced breathing period, whereas a tight correlation of mother-infant LF power was observed in older infants during this period. These results led us to infer that the effects of the mothers’ HRV on the infants’ HRV are not a merely passive phenomenon but rather an active phenomenon mediated by some internal mechanisms in infants. In other words, some computational processes in the infants’ brain and body might mediate the observed increase in the infants’ HRV via the influence of the mothers’ HRV. Probably, such mechanisms in younger infants are immature and thus take a longer time to process the mothers’ cardiac signals, resulting in the delayed increase in the infants’ HRV.

A possible explanation for this type of mechanism can be found in the predictive coding model^22^ proposed by cognitive neuroscience. From this perspective, the brain does not merely react to sensory input passively, but it constitutes an inner model to predict a future state. Perception and behaviour are actively constructed by comparing the input sense signal with the inner model prediction, then calculating their gap (predictive error) and regulating to minimise the prediction error. Recently, the principle of predictive coding has been expanded into the perception of the inner body, including heartbeat, which is called interoception^23^. Signals from the mother’s cardiac activity might reach the infant’s brain via tactile and auditory sensory modalities and might distract their inner model from the heartbeat resulting in a prediction error. In older infants with a relatively matured inner model, the beat by beat regulation of the heartbeat might be conducted to minimise the prediction error, resulting in an increase in their HRV. When the signals of the mother’s cardiac activity are terminated, reverse regulation of the inner model can quickly return the infant’s HRV to the baseline level. In younger infants, the inner model function should be premature, and the regulation of their cardiac activity based on the prediction error is delayed. Thus, the effects of mothers’ cardiac activity on infants’ HRV might appear after a necessary calculation to reduce the prediction error has been performed.

The mother’s paced breathing caused increases in HRV only in the LF power, not in the HF power. Previous studies have reported that vagal nerve activity is the main contributor to the HF component^24,25^ and LF component is not affected by respiration^26,27^, so this seems to be a specific effect of paced breathing. On the other hand, a positive correlation was found between infants’ LF power and infants’ HF power. In addition, regression analysis indicated an increase in infants’ HF power, as infants’ LF power was significant in the older group only, not in the younger group. This result implies that the amplification of mothers’ LF power due to paced breathing affected infants’ LF power through physical phenomena, and an increase in infants’ LF power caused an increase in the infants’ HF power due to inner mechanisms in infants in the older group only. Because the HF power of infants’ HRV reflects RSA, governed by efferent vagal nerve activity, this finding suggests that there is an influence of mothers’ HRV on infants’ HRV through a peripheral route and also via the efferent regulation of cardiac activity. This reasoning is consistent with the above-described interpretation from the predictive coding perspective. Among the possible routes of the influences of mothers’ HRV on infants’ HRV, the effects of mothers’ respiration should be considered along with those of mothers’ cardiac activity above described. It would be difficult to conclude that infants’ breathing itself changes due to their mother’s paced breathing, however it is possible that the mothers’ chest movements affect the infants’ HRV. Unfortunately, we were unable to accurately measure the respiration of the mothers and infants, so this question was not answerable from the present study and remains for the future.

Some limitations in this study should be recognised. Firstly, the sample size was small, although it corresponded to a previous study^5^. Thus, the findings obtained in this study should be verified and generalised using a larger sample size and a wider age range in infants. Secondly, because this study was exploratory and observational, the underlying mechanisms of the effects of mothers’ cardiac activity on infants’ cardiac activity requires further research. Additionally, this study did not measure the respiration of the mothers and infants, so, it was not possible to provide a detailed examination of the dynamics of cardiac and respiratory activities between mothers and infants. Finally, the long-lasting beneficial effects of the enhancement of infants’ HRV on mental and physical development are critical, but beyond the scope of this study. Such effects should be examined using longitudinal designs in the future.

In spite of such limitations, to the best of our knowledge, this study is the first to demonstrate that HRV in infants can be enhanced by their mothers’ HRV via direct skin-to-skin contact and that this effect is age-dependent; the reactivity of infants’ HRV depends on their developmental stage. In addition, this age-dependent reactivity of infants’ HRV could be due to the development of infants’ inner model which regulates physiological functions including cardiac activity. The present findings might be useful for developing efficient methods to enhance vagal nerve activity in infants, which would be beneficial for infants’ well-being.

## Method

### Participants

Forty dyads of healthy mothers and infants participated in this study. This sample size was determined on the basis of a related previous study^5^ (N = 11). The dyads were recruited according to the age of the infants in months, via the database of Unicharm Co. which is based in Kagawa prefecture, Japan. The infants’ ages ranged from 3 to 8 months (mean age = 5.43 months, SD = 1.26 months). There were 18 males and 22 females, with a weight range of 5.3–8.9 kg (mean weight = 7.35 kg, SD = 0.91 kg). The infants were categorised into an older group (6–8 months old; N = 19; 12 females) and a younger group (3–5 months old; N = 21; 10 females). The mother’s age was 25–41 years old (mean age = 32.63 years, SD = 4.32). Due to infants’ states (e.g. crying) and technical failures of recoding HR, 19 dyads with complete data (older group, N = 8; younger group, N = 11) were finally subjected to statistical analyses to examine mother-infant cardiac interactions. The basic attributes of the participants are provided in Table 4. We conducted this study in compliance with the Declaration of Helsinki, and all experimental protocols were approved by the Ethics Committee of Nagoya University. Written informed consent was obtained from all parents of infants before the first experimental session. Clinical trial registration details are as follows: Clinical Trial Registry Number UMIN000034772 5/11/2018 [University Hospital Medical Information Network], retrospectively registered.

### Study design

This study examined the influence of mothers’ enhanced HRV through the manipulation of their respiration on the HRV in their infants. A crossover design was adopted where all the dyads participated in both a paced-breathing session and a natural-breathing session on separate days (range of intervals; 1–20 days). The order of the breathing conditions was counter-balanced between dyads.

### Measurement of heart rate and signal processing

Small heart rate sensors (My Beat, UNION TOOL Co., Japan, 13 g, 40.8 mm × 37.0 mm × 8.9 mm) were attached on the chests of mothers and infants to record their heartbeats (R waves of electrocardiogram). As this sensor was wearable and did not have any electrical cables, it did not disturb the infant’s movements. The LF (mothers’ LF, 0.04–0.15 Hz; infants’ LF, 0.04–0.24 Hz) power and the HF (mothers’ HF, 0.15–0.4 Hz; infants’ HF, 0.24–1.04 Hz) power of each 5-minute period (pre-rest, respiration 0–5 min, respiration 5–10 min, respiration 10–15 min and post-rest) were computed using the Fast Fourier Transform. The frequency bands of the LF and HF power of infants were determined according to recent recommendations promulgated for HRV in infants^28^. These indexes were calculated using the specialised software (RRI Analyzer 2, UNION TOOL Co., Japan) for the heart rate sensor used in this study.

### Manipulation of respiration

During the paced-breathing session, mothers were instructed to breathe at a pace of six cycles per minute (cpm), to achieve a 4-second inspiratory period and 6-second expiratory period at each cycle, to enhance the LF power of HRV. The breathing pace was guided by a visual pacer on a computer monitor. During the natural-breathing session, mothers were instructed to breathe at their natural pace, as usual.

### Procedure

The experimental sessions were conducted at an experimental chamber where temperature and humidity were kept consistent (24 °C, 55%). After an examiner explained the object and methods of the experimental sessions and received consent, the mothers answered a questionnaire to collect information on their infants’ weight and developmental milestones. Prior to the start of the experiment, a heart rate sensor was attached to each mother and infant. Every infant’s diaper was changed to a new diaper before initiation of the experimental procedure, to exclude the influence of any discomfort.

The participants were allowed to rest for 5 minutes while the infants were laid on a mattress and mothers sat on the floor. After the five minutes pre-rest period, mothers held their infants in their arms and sat on a chair. The mothers were instructed to breathe at a pace of 6 cpm (paced-breathing session) or at a natural pace (natural-breathing session) for 15 minutes. While the mothers were holding their infants in their arms, their bodies were in full contact throughout the manipulation of respiration to induce mutual physical influences. After this respiration period, participants were instructed to rest for 5 minutes again (post-rest period) in the same way as the pre-rest period.

The behaviour of mothers and infants were recorded by a video camera to check for any problems during the experimental session. After each experimental session, mothers were asked to answer a questionnaire by mail about their infant’s profile and health status at birth. According to the results of the questionnaire, the participants were healthy mothers and infants without any abnormalities at birth and were not receiving treatment for disease.

### Transfer entropy

TE is a non-parametric statistic, measuring the direct transfer of information between two time-series processes, calculated using the following formula. In other words, TE reflects the magnitude of one time-series process’ influence on the other.

$${T}_{Y\to X}=\sum _{{x}_{t+1},{x}_{t}\in X}\sum _{{y}_{t}\in Y}p({x}_{t+1},{x}_{t},{y}_{t})\log \,\frac{p({x}_{t+1}|{x}_{t},{y}_{t})}{p({x}_{t+1}|{x}_{t})}$$

where *Y* and *X* are probabilistic functions and *y*_*t*_ and *x*_*t*_ are values of the component of Y and X at a time point *t*. In this study, the TE between the heartbeats of mother and infant was calculated using the following formula, for two directions: from mother to infant and infant to mother. When calculating TE, the fixed bin approach was used to estimate the probabilities in the above formula, by allocating the data points to bins of 200 msec. Thus, *y*_*t*_ and *x*_*t*_, mean occurrence/non-occurrence of a peal of R wave, indicated 1 or 0. TE was calculated at each 5-minute period (pre-rest, respiration 0–5 min, respiration 5–10 min, respiration 10–15 min and post-rest) during the experimental procedure. As the TE scores were sometimes small, the logged TE values were subjected to statistical analyses.

### Statistical analysis

Repeated measures ANOVAs with a between-participant factor of developmental stage (older, younger) and two within-participant factors of condition (natural-breathing, paced-breathing) and periods (pre-rest, respiration 0–5 min, respiration 5–10 min, respiration 10–15 min and post-rest), were performed to analyse the LF and HF powers of HRV in mothers and infants, separately. To obtain the logged values of heartbeat TE, the same repeated measures ANOVAs were performed, to assess the direction of influences from mother to infant and from infant to mother, separately. The Greenhouse-Geisser correction was applied when a violation of sphericity was observed. Post-hoc analyses were performed by Bonferroni tests (*p* < 0.05). Partial eta-squared was computed as the effect size.

A series of regression analyses was conducted to examine associations between the mothers’ HRV and the infants’ HRV in the younger and older groups. We firstly performed exploratory correlation analyses between the LF power of mothers, the LF power of infants and the HF power of infants. With reference to results of the correlation analyses, we chose changes in mothers’ LF power during the 0–5 min, 5–10 min and 10–15 min periods as possible independent variables and the change in infants’ LF power during the 0–5 min, 5–10 min, 10–15 min and post-rest periods as possible dependent variables to perform a regression analysis. We also conducted a regression analysis to examine associations between the infants’ HF power and mothers’ LF power. Furthermore, we conducted a regression analyses to examine different associations between each individual infants’ HF power and LF power. We chose changes in infants’ HF power during the 0–5 min, 5–10 min and 10–15 min periods as possible dependent variables and changes in mothers’ and infants’ LF power during the 0–5 min, 5–10 min and 10–15 min as possible independent variables. The significance (*p* < 0.05) of the interaction term between each mean-centred independent variable and the moderator (developmental stage) was tested with each combination of independent-dependent variables. Data without technical problems from 22 mothers and 30 infants were subjected to analyses. Complete data from 19 dyads of mothers and infants were used for the ANOVA for TE and regression analyses. The basic attributes of the participants are presented in Table 4.

**Table 4:** Basic characteristics of the participants.

			N	Infant’s sex		Mother’s age		
				Male	Female	Age (years)	Mean	SD
A		40 dyads	40	18	22	25–41	32.63	4.32
B		22 mothers	22	9	13	25–41	32.77	4.40
C		30 infants	30	14	16	25–41	33.07	4.22
	Younger	16 infants	16	8	8	25–41	33.69	4.08
	Older	14 infants	14	6	8	26–40	32.36	4.41
D		19 dyads	19	8	11	25–41	32.84	4.35
	Younger	11 infants	11	5	6	25–41	33.36	4.54
	Older	8 infants	8	3	5	29–40	32.13	4.26

A: Recruited participants, B: mothers with complete data, C: infants with complete data and D: dyads with complete data.

## Supplementary information

Supplementary information .

Supplementary Dataset.

## Data Availability

All data analysed during this study are included in this published article (and its supplementary information files).
