# Supplementary material for: Cardiac interaction between mother and infant: enhancement of heart rate variability
Source: Sci Rep. 2019 Dec 27;9:20019. doi: 10.1038/s41598-019-56204-5 (PMC6934483; doi:10.1038/s41598-019-56204-5)
Supplement: Supplementary file 1 — Supplementary information . [file 41598_2019_56204_MOESM1_ESM.docx]

**Supplementary Information for Manuscript: Cardiac interaction between mother and infant: enhancement of heart rate variability**

**Authors:** Ayami Suga^1,2^*, Maki Uraguchi^2^, Akiko Tange^1^, Hiroki Ishikawa^1^ & Hideki Ohira^2^

^1^ Unicharm Corporation, 769-1602 Kagawa, Japan

^2^ Department of Psychology, Nagoya University, 464-8601 Nagoya, Japan

* ayami-suga@unicharm.com

**Supplementary　Table　S1. Descriptive statistics (Mothers)**

The table is based on the complete data from 22 mothers.

LF; 0.04-0.15Hz, HF; 0.15-0.4Hz

**Supplementary　Table　S2. Descriptive statistics (Younger infants)**

The table is based on the complete data from 16 Younger infants.

LF; 0.04-0.24Hz, HF; 0.24-01.04Hz

**Supplementary　Table　S3. Descriptive statistics (Older infants)**

The table is based on the complete data from 14 older infants.

LF; 0.04-0.24Hz, HF; 0.24-01.04Hz
